# Impact of testosterone-based gender-affirming hormone therapy on toll-like receptor transcript levels and peripheral blood leukocyte counts in transmasculine individuals

**DOI:** 10.3389/fimmu.2026.1752956

**Published:** 2026-03-18

**Authors:** Hüseyin Cihan, Özge Güngör, Gökcen Ünal Kocabas, Esma Pehlivan Köroglu, Kübra Gülpinar, Erhan Pariltay, Ömür Ardeniz, Guy T’Sjoen, Jonatan Leffler, Bettina Winzeler, Banu Sarer Yürekli

**Affiliations:** 1 Faculty of Medicine, Zurich University, Zurich, Switzerland; 2 Zurich University Hospital, Zurich, Switzerland; 3 Innovation Focus Gender Variance, Basel University Hospital, Basel, Switzerland; 4 Faculty of Medicine, Ege University, Izmir, Türkiye; 5 Department of Medical Genetics, Ege University Hospital, Izmir, Türkiye; 6 Department of Endocrinology, Ege University Hospital, Izmir, Türkiye; 7 Department of Allergy and Immunology, Ege University Hospital, Izmir, Türkiye; 8 Department of Endocrinology, Ghent University Hospital, Ghent, Belgium; 9 The Kids Research Institute Australia, University of Western Australia, Perth, WA, Australia

**Keywords:** GAHT, testosterone, TLRs, transgender, transmasculine, leukocyte

## Abstract

**Background:**

Sex differences in immune responses are partly attributed to sex hormones, with testosterone generally associated with dampened immune responses. However, the specific impact of testosterone on the innate immune system in humans remains unclear. We examined changes in toll-like receptor (TLR) transcript levels and peripheral blood leukocyte parameters during masculinizing hormone therapy in transmasculine individuals.

**Methods:**

We conducted a 6-month prospective observational study in 20 transmasculine individuals initiating testosterone-based gender-affirming hormone therapy. Peripheral blood mononuclear cells were collected at baseline and after 6 months of therapy. Gene transcript levels of TLR1–8, TLR10, MD2, and CD14 were quantified by RT-qPCR. Serum hormones and hematologic parameters were measured by standard assays.

**Results:**

After 6 months of testosterone therapy, serum testosterone levels rose to values comparable to the range in cisgender men. Lymphocyte and monocyte counts increased (*p* = 0.02 and *p* = 0.006, respectively). TLR8 and TLR10 mRNA levels were increased relative to baseline (*p* = 0.003 and *p* = 0.001, respectively). TLR2 and MD2 transcript levels showed nominal declines (unadjusted *p* < 0.05) but did not remain significant after correction. No other TLRs showed significant changes. Exploratory correlation analyses show that the magnitude of estradiol decline and monocyte expansion was associated with changes in TLR1, TLR2, and TLR8 transcript levels.

**Conclusions:**

In this cohort, testosterone-based gender-affirming hormone therapy is associated with the upregulation of TLR8 and TLR10 transcripts, suggesting that sex hormone shifts (increased testosterone and decreased estradiol) may modulate certain innate immune receptors at the transcript level. These findings provide insights into how gender-affirming hormone therapy can influence the innate immune system in transmasculine individuals.

## Introduction

Sex hormones influence immune responses, susceptibility to infectious diseases, and autoimmune conditions (1, 2). Toll-like receptors (TLRs) are components of the innate immune system that detect pathogen-associated molecular patterns (PAMPs) and trigger inflammatory signaling cascades (3). Testosterone has been demonstrated to modulate immune function and inflammation. Some of these effects may be mediated by changes in immune receptor gene expression (e.g., TLR transcript levels) (4).

Testosterone-based gender-affirming hormone therapy (GAHT) in transmasculine individuals induces a range of systemic physiological changes, including suppression of endogenous estrogen production, stimulation of erythropoiesis, alterations in lipid metabolism, and increased muscle mass, mediated primarily through activation of androgen receptors (5–7). Despite its broad physiological effects, the impact of the immune system in transmasculine individuals is less known.

TLR expression levels show sex-related differences influenced by both sex hormones and number of sex chromosomes (8–10). A mouse study demonstrated that TLR4 expression in macrophages increases following orchiectomy and that subsequent exposure to testosterone suppresses TLR4 levels in androgen-deprived macrophages (11). Other studies have also reported that male mice express higher levels of TLR2, while higher levels of TLR4 are shown on macrophages in female mice (12). Conversely, other studies have also found no discernible sex differences in TLR4 mRNA expression under baseline conditions (13, 14). In humans, peripheral blood mononuclear cells (PBMCs) treated with estradiol showed increased expression of TLR8 at both the mRNA and protein levels, but exposure of human PBMCs to testosterone did not affect the expression of TLR2, TLR3, TLR4, TLR7, TLR8, and TLR9 (15, 16). The expression of TLR7 and TLR8 is further modulated by their gene localization on the X chromosome and their potential to escape X chromosome inactivation (XCI). TLR7 and TLR8, two X-linked receptors, may both escape XCI in human immune cells. Single-cell analyses have shown higher frequencies of cells co-expressing TLR7 and TLR8 in individuals with two X chromosomes, such as cisgender women (XX) and Klinefelter syndrome men (XXY), compared to cis men (XY) (17, 18). Recent studies have begun to show immune system adaptations during testosterone therapy in transmasculine individuals receiving testosterone-based GAHT following TLR7/8 stimulation, and plasmacytoid dendritic cells (pDCs) showed reduced type I interferon (IFN-I) responses following administration of testosterone-based GAHT (19). Consistent findings were observed in a separate cohort of transmasculine individuals undergoing testosterone-based GAHT; following TLR7/8 stimulation, single-cell RNA sequencing of pDCs revealed decreased induction of interferon-stimulated genes and attenuated hallmark IFN-I transcriptional pathways. Furthermore, stimulation of monocytes with the TLR4 agonist, lipopolysaccharide (LPS), showed further potentiated monocyte TNF responses following testosterone-based GAHT (20).

Although previous studies have revealed important insights into the immunological impact of testosterone-based GAHT, including changes in immune cell profile (21) and immune cell function, the effects of testosterone-based GAHT on TLR receptor transcript levels remain underexplored. The aim of this study was to evaluate longitudinal changes of TLR transcript levels before and after 6 months of testosterone-based GAHT in transmasculine individuals.

## Methods

### Study objectives

This study had three main objectives: first, to characterize longitudinal changes in whole-blood CD14, MD-2, TLR1–8, and TLR10 mRNA transcription in transmasculine individuals initiating testosterone-based GAHT; second, to assess changes in sex hormones and hematologic indices (including leukocyte subsets and hemoglobin) over the same period; and third, to explore how changes in estradiol and testosterone, and in blood leukocyte subsets, relate to changes in TLR transcript levels, with a particular focus on receptor-specific patterns rather than uniform up- or downregulation of innate immune sensing.

### Study design and participants

We conducted a prospective paired, pre–post study in transmasculine individuals initiating testosterone-based GAHT. Twenty participants were enrolled at the Department of Endocrinology, Ege University Hospital, Izmir, Türkiye between October 2022 and November 2023 and provided blood samples at baseline (month 0) and after 6 months of GAHT. Inclusion criteria were age ≥18 years, eligibility for GAHT per clinical guidelines, and no acute infection within 2 weeks. Exclusion criteria were current immunosuppressive therapy, chronic inflammatory disease flare, or malignant disease. The study was approved by the Ege University KAEK 22-9.2/2, and all participants provided written informed consent.

### Gender-affirming hormone therapy

Testosterone was prescribed per routine clinical practice and individuals received SUSTANON (testosterone esters) at 250 mg every 3 weeks. Dose adjustments followed World Professional Health for Transgender Health, Standard of Care-8 (5, 22). Concomitant medications were also recorded.

### Peripheral blood collection and clinical laboratory assays

Peripheral blood was obtained at baseline and after 6 months of treatment (fasting morning samples when possible). Complete blood count (WBC, differential, hemoglobin, MCV, platelet, and MPV) was measured on an automated analyzer (SYSMEX XN-2100). Serum FSH, LH, estradiol, and total testosterone were quantified using Roche diagnostic e801 with the laboratory’s reference ranges and internal quality control procedures. Paired hormone data were not available for all participants (FSH and LH: *n* = 12 pairs; estradiol: *n* = 14 pairs); analyses involving these hormones used the maximum available paired samples.

### Whole-blood RNA extraction, cDNA synthesis, and RT-qPCR

Whole blood (≈0.25 mL) was homogenized in RiboEx™ LS (phenol–guanidinium), phase-separated with chloroform, and RNA was isolated from the aqueous phase purified on silica spin columns (EzPure™). RNA yield/quality were checked on NanoDrop 1000 (accepted: A260/280 = 1.8–2.0, A260/230 ≥ 1.7, ≥40 ng/µL). cDNA was synthesized using iScript™ (Bio-Rad). RT-qPCR was performed on a LightCycler^®^ 480 with SYBR Green in technical triplicates. Melt curves confirmed single products; efficiencies, 90%–110%. Reactions with Ct > 35, non-specific melts, or replicate SD > 0.5 Ct were excluded; no-template and no-RT controls were also included in each assay.

### Transcript level calculations

For each target, ΔCt = Ct(target) − mean Ct(housekeepers). Change over time was ΔΔCt = ΔCt6m_{6m}6m − ΔCt0_{0}0. For interpretability, log2 fold change was defined as log2FC = −ΔΔCt (positive values indicate higher transcript levels at 6 months).

### Statistical analysis

Continuous variables are summarized as mean ± SD and median (IQR). Paired comparisons between 0 and 6 months used paired *t*-tests (approximately normal differences) or Wilcoxon signed-rank tests otherwise; two-sided α = 0.05. For the immune marker panel (TLR1–8, TLR10, MD2, and CD14), Benjamini–Hochberg false discovery rate (FDR) correction was applied across genes. Analyses were performed in R 2024.12.0 with packages *stats*, *dplyr*, and *ggplot2*. Figures include paired box plots for selected genes and a forest plot of mean log2FC with 95% bootstrap CIs. Spearman rank correlations were computed between the Δ (6 months − baseline) values of each hormone, blood cell count, and TLR transcript levels. Spearman correlations between changes (Δ values) were treated as exploratory. A two-sided α = 0.05 was used for these correlations without formal adjustment for multiple comparisons, given the hypothesis-generating intent.

## Results

### Participants and baseline

A total of 20 transmasculine individuals were enrolled and provided paired samples at baseline and 6 months. Mean age was 27.1 ± 4.5 years [median 26.5 (23.0–29.0)]; baseline laboratory indices are summarized in **Table 1**. Paired immune-marker (RT-qPCR) data were available for all 20; paired hormonal data were complete for testosterone (*n* = 20) and partially missing for FSH and LH (each *n* = 12 pairs) and estradiol (*n* = 14 pairs).

**Table 1:** Changes in laboratory parameters from baseline to 6 months.

Variable	Baseline (mean ± SD)	6 Months (mean ± SD)	*p* (*t*-test)
FSH (IU/L)	5.27 ± 1.76	5.41 ± 2.73	0.88
LH (IU/L)	11.09 ± 7.76	7.70 ± 5.20	0.12
Estradiol (pg/mL)	111.7 ± 75.6	65.6 ± 35.2	0.04
Testosterone (ng/dL)	36.9 ± 12.2	550.7 ± 135.2	<0.0001
WBC (×10^9^/L)	7.81 ± 1.61	7.93 ± 1.11	0.74
Neutrophil (×10^9^/L)	4.77 ± 1.26	4.69 ± 1.10	0.79
Lymphocyte (×10^9^/L)	2.15 ± 0.53	2.41 ± 0.48	0.02
Monocyte (×10^9^/L)	0.52 ± 0.09	0.58 ± 0.08	0.006
Hemoglobin (g/dL)	12.67 ± 1.06	14.13 ± 1.43	0.0003
MCV (fL)	87.9 ± 5.6	84.6 ± 5.9	0.003
MPV (fL)	10.4 ± 0.7	10.3 ± 0.8	0.44
Platelet (×10^9^/L)	304 ± 81	313 ± 95	0.59

### GAHT induces changes in blood count parameters

**Figure 1:**
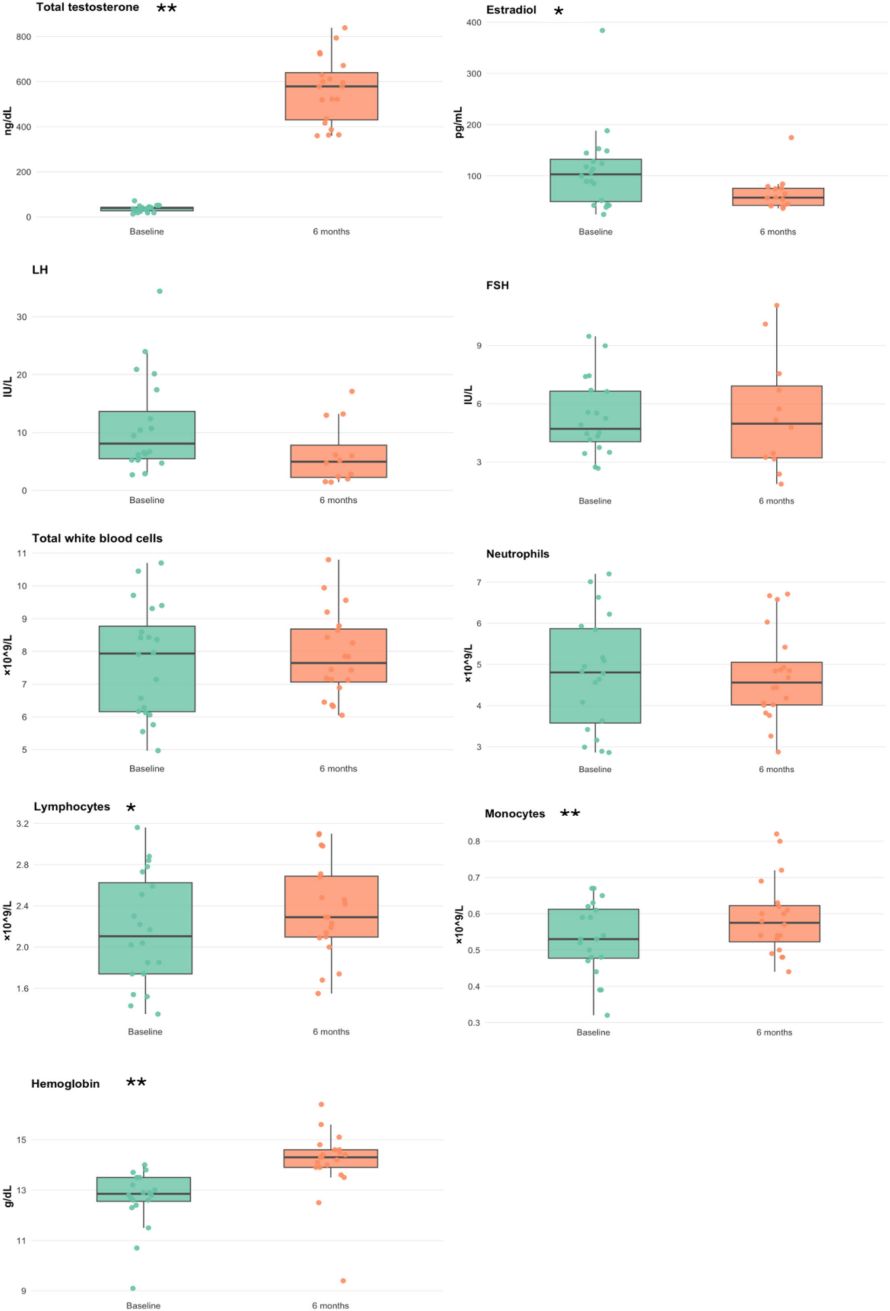
Hematological parameters at baseline and 6 months after masculinizing GAHT (testosterone-based therapy). Box plots display the median and interquartile range (IQR), with individual data points. Statistically significant differences indicated by the corresponding significance markers (*p < 0.05, **p < 0.01). LH, luteinizing hormone; FSH, follicle-stimulated hormone.

To assess the impact of testosterone-based GAHT, hormone and blood counts were evaluated at both time points. Total testosterone increased, accompanied by a decrease in estradiol, while LH and FSH showed no significant change. Among blood counts, lymphocyte and monocyte counts as well as hemoglobin increased, whereas mean corpuscular volume decreased (**Figure 1**). WBC, neutrophils, MPV, and platelets showed no significant change (all *p* > 0.05). Full summaries and *p*-values are provided in **Table 1**.

### GAHT induces changes in TLR8 and 10 transcript levels

**Figure 2:**
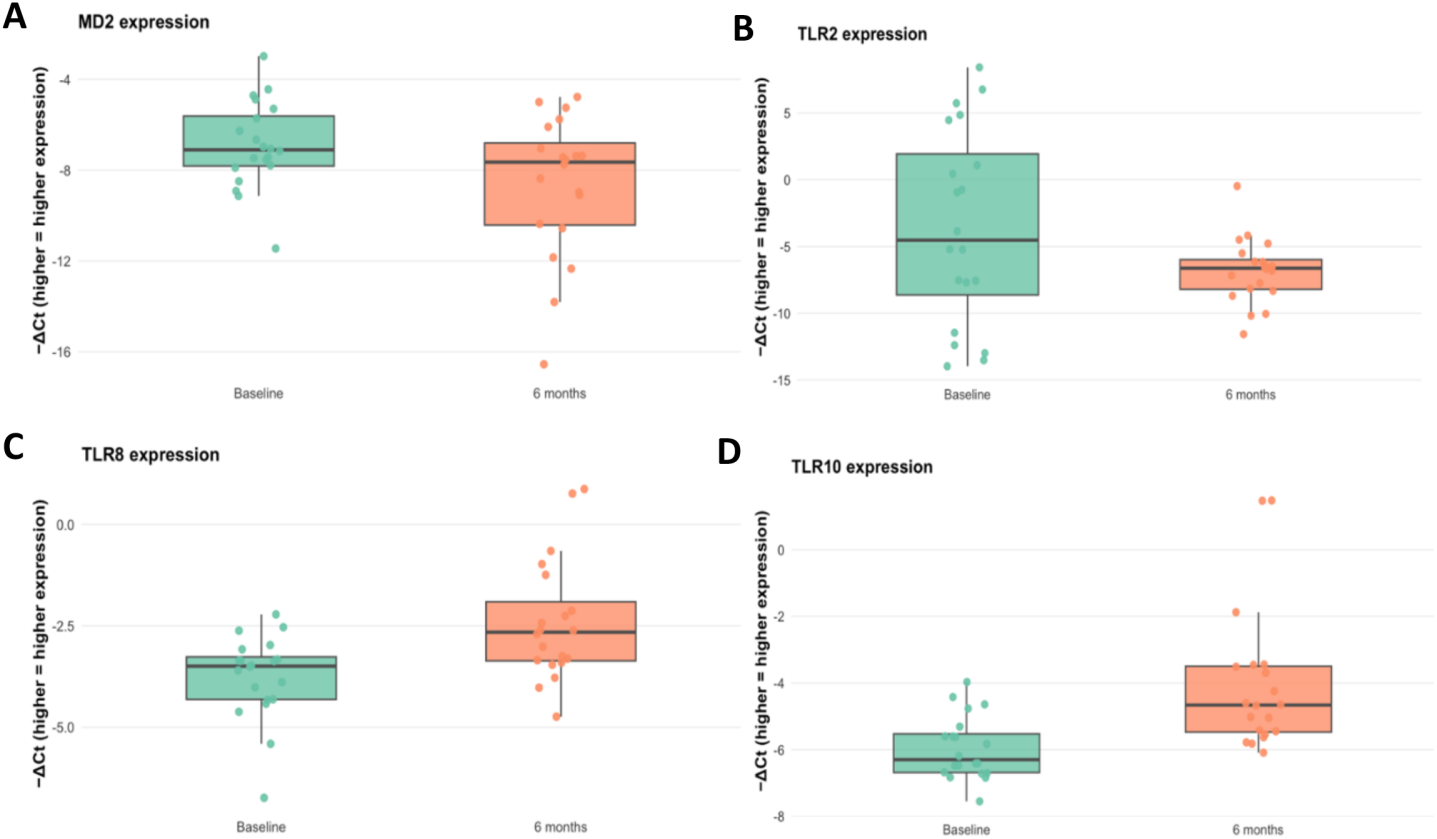
Expression of **(A)** MD2, **(B)** TLR2, **(C)** TLR8, and **(D)** TLR10, presented as ΔCt values, at baseline and 6 months after initiation of masculinizing GAHT (testosterone-based therapy). Box plots indicate the median and interquartile range (IQR), and dots represent individual participants. Significant changes between baseline and 6 months were observed for MD2 (*p* = 0.031), TLR2 (*p* = 0.046), TLR8 (*p* = 0.003), and TLR10 (*p* = 0.001) (paired t-test).

**Figure 3:**
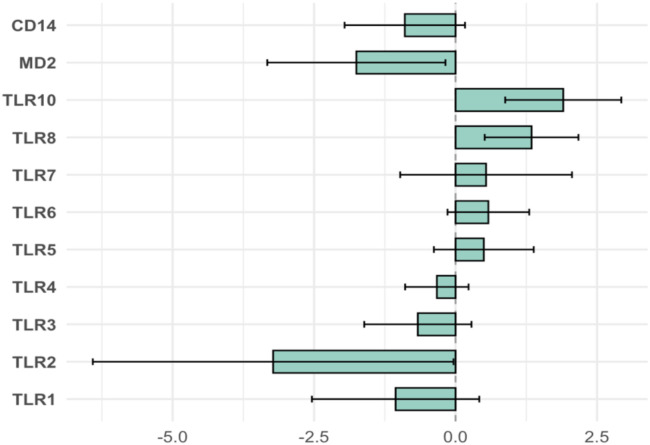
Immune transcript changes after 6 months of testosterone GAHT (*n* = 20). Bars show mean log_2_ fold change (log_2_FC = −ΔΔCt) for TLR1–7, TLR8, TLR10, MD2/LY96, and CD14; positive = higher transcript level at 6 months. Error bars are 95% bootstrap CIs; dashed line indicates no change.

To evaluate the impact of testosterone-based GAHT on TLR transcript levels, we quantified RNA transcripts of TLR1–8, TLR10, MD2, and CD14 in whole blood at baseline and 6 months. Two receptors showed a significant increase in transcript level: TLR8 (*p* = 0.003) and TLR10 (*p* = 0.001) at 6 months (**Figures 2C, D**). MD2 showed a decrease in transcript level (*p* = 0.031) (**Figure 2A**) and TLR2 displayed a decreasing trend (**Figure 2B**) (*p* = 0.048); TLR1 and TLR3–7 showed no significant change (all *p* > 0.10) (**Supplementary Figure**). After Benjamini–Hochberg FDR correction across the 11 immune components (MD2, CD14, TLR1–8, and TLR10), TLR10 and TLR8 remained statistically significant (*q* < 0.05), whereas TLR2, MD2, and others did not. For interpretability, mean −ΔΔCt with 95% bootstrap CIs were also displayed as a forest plot for all relevant targets (**Figure 3**).

### Changes in TLR transcript levels are associated with hormone changes

**Figure 4:**
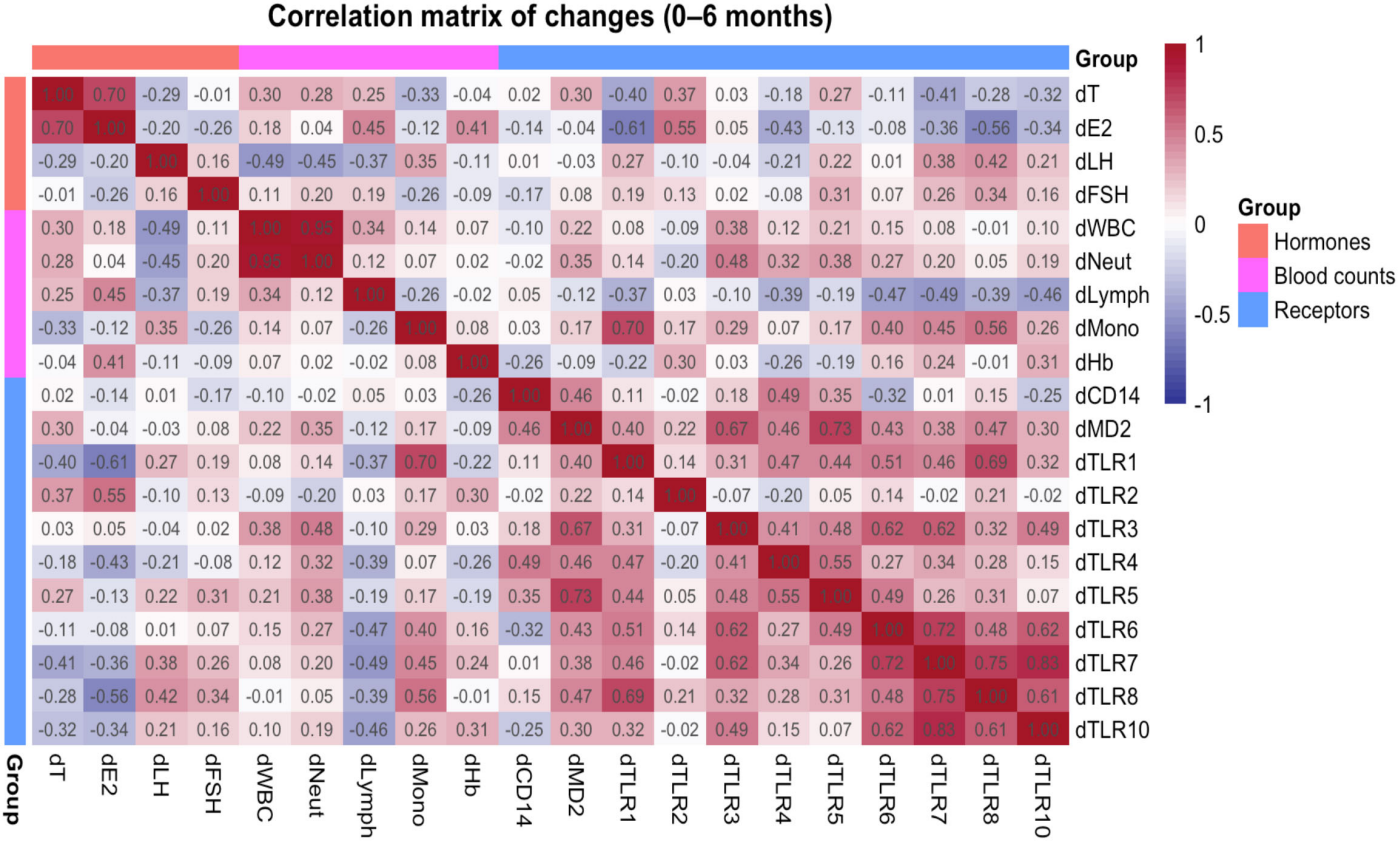
Correlation matrix of 0- to 6-month changes in hormones, blood cell counts, and TLR transcript levels. Spearman correlation coefficients (ρ) between paired 0- to 6-month changes (Δ) are shown for sex hormones (Δtotal testosterone, Δestradiol, ΔLH, and ΔFSH), blood parameters (Δtotal white blood cells, Δneutrophils, Δlymphocytes, Δmonocytes, and Δhemoglobin) and innate immune transcripts (ΔCD14, ΔMD-2, and ΔTLR1–ΔTLR10). Each cell displays the Spearman ρ for the corresponding variable pair. The color scale indicates the direction and magnitude of the correlation (red = positive, blue = negative; white ≈ no correlation), with darker shades representing stronger associations. Variables are grouped by type (hormones, blood counts, and receptors) along the axes.

To explore how hormonal and hematologic changes related to innate receptor dynamics, exploratory Spearman correlations were performed between 0- and 6-month deltas (Δ) in hormone concentrations, immune cell counts, and TLR mRNA levels (**Figure 4**) among all pairs tested. Because of missing hormone data, correlation analyses involving estradiol, FSH, or LH include fewer than 20 pairs (see Methods). First, the change in estradiol (ΔE2, defined as 6 months minus baseline) was correlated with the change in TLR2 transcript level (ΔTLR2; ρ = 0.55, *p* = 0.04, *n* = 14). In contrast, ΔE2 was negatively correlated with both ΔTLR1 (ρ = −0.61, *p* = 0.02, *n* = 14) and ΔTLR8 (ρ = −0.56, *p* = 0.04, *n* = 14). A cell–receptor association was also observed: the change in monocyte count (Δmonocytes) correlated with ΔTLR1 (ρ = 0.70, *p* < 0.001, *n* = 20). Finally, the change in receptors TLR7 and TLR8 showed a strong positive correlation between their Δ values (ρ = 0.75, *p* < 0.001, *n* = 20).

## Discussion and conclusion

In this longitudinal cohort of transmasculine individuals, 6 months of testosterone-based GAHT associated with significant shifts in some innate immune transcripts and hematologic parameters. We observed significant upregulation of TLR8 and TLR10 mRNA; after Benjamini–Hochberg correction, other TLRs (TLR1–7), MD2, and CD14 showed no statistically significant change. These changes coincided with the expected hormonal and hematologic effects of testosterone therapy, a large rise in serum testosterone and a decline in estradiol, a marked increase in hemoglobin with a decrease in mean corpuscular volume, and increases in lymphocyte and monocyte counts.

The increase in TLR8 and TLR10 transcript level is of particular interest. Interestingly, our finding of increased TLR8 transcript levels despite a decline in estradiol appears paradoxical given prior evidence. TLR8 is encoded on the X chromosome and has been identified as an estrogen-responsive gene (15). In female immune cells, 17β-estradiol (E2) upregulates TLR8 via ERα binding, and TLR8 expression and responses are higher in women. By contrast, one *in vitro* study found that exposing human PBMCs to testosterone did not alter TLR8 (or other TLR) transcripts (8). Additionally, both androgen receptor signaling and X-chromosome gene dosage (partial escape from X inactivation) could modulate TLR8 transcription in ways that differ from acute *in vitro* hormone exposure (17). It is possible that the chronic hormonal milieu during GAHT (high testosterone with reduced but not absent estradiol) or shifts in immune cell composition alter TLR8 regulation. Indeed, we observed a positive (though non-significant) correlation between the increase in monocyte count and the increase in TLR8 transcripts, consistent with the notion that a higher fraction of TLR8-expressing monocytes could contribute to the bulk TLR8 mRNA increase. However, this explanation remains speculative, and the discrepancy between the expected estradiol effect and our findings highlights a gap in understanding. Functional consequences of this shift remain to be determined.

Although much less is known about TLR10, emerging data suggest that it may act as an inhibitory or anti-inflammatory receptor. In particular, TLR10 signaling has been shown to reduce IL-1β production and increase IL-1 receptor antagonist (IL-1Ra) levels (23, 24). Thus, higher TLR10 transcript level after testosterone could reflect a shift toward dampening of innate inflammation.

TLR2 (together with TLR1 or TLR6) is central to sensing bacterial lipoproteins and Gram-positive components, while MD2 is the essential co-receptor for TLR4 in LPS detection. In our cohort, TLR2 and MD2 transcript levels showed nominal decreases, but these did not remain statistically significant after FDR correction. Accordingly, we interpret these findings as hypothesis-generating, and we cannot infer altered pathogen-sensing capacity or TLR4 responsiveness from transcript data alone. Although MD2 is required for LPS recognition by the TLR4–MD2 complex, establishing functional consequences would require complementary protein-level quantification and stimulation assays (e.g., LPS-induced cytokine outputs).

The other TLRs (TLR1, TLR3, TLR4, TLR5, TLR6, and TLR7) and CD14 showed no significant change. This lack of change may indicate that short-term GAHT does not uniformly alter all innate sensors, or that opposing hormonal effects balanced out. For example, although data from experimental models suggest that testosterone reduces TLR4 expression on macrophages (11, 12), we did not detect a change in TLR4 mRNA in human PBMCs. Differences in target cells (murine peritoneal macrophages vs. human PBMCs) and timeframe (acute vs. 6 months of therapy) are likely to account for this. It may also be that protein-level regulation (e.g., receptor trafficking or co-receptor availability) is more sensitive than mRNA.

Overall, these patterns only partially reflect known sex differences in immunity. It is well studied that cis women generally have stronger immune responses; for example, TLR expression levels may vary by sex, with TLR3 and TLR7 tending to exhibit higher expression in women and TLR2 and TLR4 having been reported to be increased in men, yet these patterns can differ across cell subpopulations (25–27). Notably, although circulating estradiol levels decreased under testosterone-based GAHT, estradiol levels remained higher compared to cisgender men reference levels and even positively correlated with testosterone possibly due to aromatization; this effect should not be overlooked when interpreting these findings. It is also worth noting that genetic factors (e.g., X-chromosome number and microRNAs) are likely to contribute to sex bias independently of hormones (9, 28).

Over 6 months of testosterone therapy, estradiol and several TLR transcripts showed correlation. Within-person changes revealed that ΔE2 correlated positively with ΔTLR2 but negatively with ΔTLR1 and ΔTLR8, which may suggest that estradiol loss is accompanied by the downregulation of TLR2, the relative preservation of TLR1, and the upregulation of TLR8. In addition, participants with greater monocyte expansion tended to have less TLR1 decline and more TLR8 increase, suggesting that changes in cell-subset composition could contribute to the TLR results. In other words, the observed TLR8 upregulation might be partly due to a higher proportion of monocytes (which abundantly express TLR8) in the blood at 6 months, rather than an intrinsic increase of TLR8 per cell. In line with our correlation analysis, serum testosterone and estradiol levels were positively associated, likely reflecting aromatization of testosterone to estradiol. Therefore, these findings are better interpreted as effects of the overall androgen–estrogen milieu rather than a direct effect of testosterone alone. Furthermore, baseline estrogen concentrations may differ depending on the menstrual cycle phase; hence, future research should carefully consider baseline estrogen levels in their analyses. Notably, these correlations were exploratory and were not corrected for multiple comparisons, so they should be interpreted with caution as associations.

Beyond transcription, testosterone had expected effects on blood cells. The rise in hemoglobin and hematocrit is a well-known androgenic effect, and testosterone stimulates erythropoiesis via erythropoietin and iron metabolism. Our observed hemoglobin increases align with existing literature in both hypogonadal men and transmasculine individuals on testosterone (29–31). In addition, we saw increases in lymphocyte and monocyte counts. A study on cisgender men showed that graded testosterone dosing significantly increased neutrophil and monocyte counts (and total leukocytes) while having negligible effect on lymphocytes (32). They concluded that testosterone promotes myelopoiesis. Our finding of higher monocytes and lymphocytes is largely in line with this myeloid priming effect, although their study found no significant changes in absolute lymphocytes, whereas we did. This difference could reflect differences in cohort (young transmasculine individuals vs. older hypogonadal men) or sample size (21).

Taken together, these data show that sex hormones are linked to dynamic changes in the immune system, and estradiol withdrawal during transmasculine hormone therapy may be associated with these shifts.

Although our data suggest that GAHT induces changes to transcript level of TLRs, there are several limitations to the study. These include the fact that only mRNA was measured, and changes in transcript levels may not directly translate to functional protein changes or altered cell behavior, and without functional assays (e.g., cytokine response to TLR ligands), we cannot conclude that GAHT modulates immune responses, only that it alters certain immune receptor transcripts. Furthermore, RNA was extracted from whole blood, limiting the ability to identify cell-specific expression as well as to adjust for changes in immune cell composition. Thus, we cannot fully distinguish cell-intrinsic expression changes from shifts in leukocyte composition. Another limitation is the potential impact of X-chromosome dosage; however, no X-chromosome-specific analyses (e.g., assessment of X-inactivation patterns or copy-number variation) were performed as part of this study. All participants had been previously genetically confirmed to have a two-X chromosome complement prior to inclusion, and individuals with known intersex variations were excluded before referral to the endocrinology clinic.

Additionally, our study did not include a separate control group of individuals not on GAHT (e.g., cisgender men or women) and the sample size was small (*n* = 20), which limits statistical power and generalizability especially with potential confounders (e.g., age and lifestyle), and follow-up time was relatively short (note that some immunological effects might require longer exposure to testosterone therapy), although both were similar to other recent studies in the field (20, 21). Lastly, hormone–TLR correlations were exploratory and reported with nominal *p*-values (no adjustment for multiple comparisons); therefore, they should be interpreted cautiously.

In summary, 6 months of testosterone-based GAHT in transmasculine individuals was associated with selective changes in innate immune transcripts and expected shifts in blood counts. Notably, TLR8 and TLR10 transcript levels increased, highlighting that sex hormone alterations can influence specific innate immune sensors. These focused transcript changes indicate a complex, bidirectional influence of sex hormones on the immune system. Future studies should explore the functional outcomes of these shifts, including protein-level changes, cytokine responses, and clinical correlates. Such research will deepen our understanding of sex hormone–immune crosstalk and inform the care of transgender and gender-diverse populations.

## Data Availability

The raw data supporting the conclusions of this article will be made available by the authors, without undue reservation.
