# Supplementary material for: Impact of testosterone-based gender-affirming hormone therapy on toll-like receptor transcript levels and peripheral blood leukocyte counts in transmasculine individuals
Source: Front Immunol. 2026 Mar 18;17:1752956. doi: 10.3389/fimmu.2026.1752956 (PMC13038929; doi:10.3389/fimmu.2026.1752956)
Supplement: Supplementary file 1 [file DataSheet1.pdf]

Supplementary Material

PCR Primers sequences

**CD14 Human qPCR Primer Pair (NM_000591)**	**F:CTGGAACAGGTGCCTAAAGGAC**
	**R:GTCCAGTGTCAGGTTATCCACC**
**MDM2 Human qPCR Primer Pair (NM_002392)**	**F:GCCATGCCTGCCCACTTTAG**
	**R:CCAGGCTGCCATGTGACCTA**
**TLR1 Human qPCR Primer Pair (NM_003263)**	**F:CAGCGATGTGTTCGGTTTTCCG**
	**R:GATGGGCAAAGCATGTGGACCA**
**TLR2 Human qPCR Primer Pair (NM_003264)**	**F:CTTCACTCAGGAGCAGCAAGCA**
	**R:ACACCAGTGCTGTCCTGTGACA**
**TLR3 NM_003265**	**F:GTGCCGTCTATTTGCCACACA**
	**R:GCACACAGCATCCCAAAGGG**
**TLR4 Human qPCR Primer Pair (NM_138554)**	**F:AGACGGTGATAGCGAGCCAC**
	**R:TTAGGGCCAAGTCTCCACGC**
**TLR5 Human qPCR Primer Pair (NM_003268)**	**F:ACGTGGCTTCTCCACAGTCA**
	**R:CGGACAGCGCCAACATTCTC**
**TLR6 Human qPCR Primer Pair (NM_006068)**	**F:CCTTTCTCTTCCTCCTGAAAGCA**
	**R:TGGTCATGATGTTGCAGTGGC**
**TLR7 Human qPCR Primer Pair (NM_016562)**	**F:CTTTGGACCTCAGCCACAACCA**

	**R:CGCAACTGGAAGGCATCTTGTAG**
**TLR8 Human qPCR Primer Pair (NM_016610)**	**F:ACTCCAGCAGTTTCCTCGTCTC**
	**R:AAAGCCAGAGGGTAGGTGGGAA**
**TLR 10 Human qPCR Primer Pair (NM_030956)**	**F:TGTGGGCTTTTCTGGGCAAAC**
	**R:TGGTCCCCAACTTCCCAAGG**
**actin beta (ACTB) NM_001101.5**	**F:ACAGAGCCTCGCCTTTGCC**
	**R:CGAGCGCGGCGATATCATCA**
**GAPDH (KONTROL). NM_001357943.2**	**F:GTCTCCTCTGACTTCAACAGCG:**
	**R:ACCACCCTGTTGCTGTAGCCAA**

TLR transcript levels

Lower ΔCt indicates higher transcript level, decreases in ΔCt reflect up-regulation.

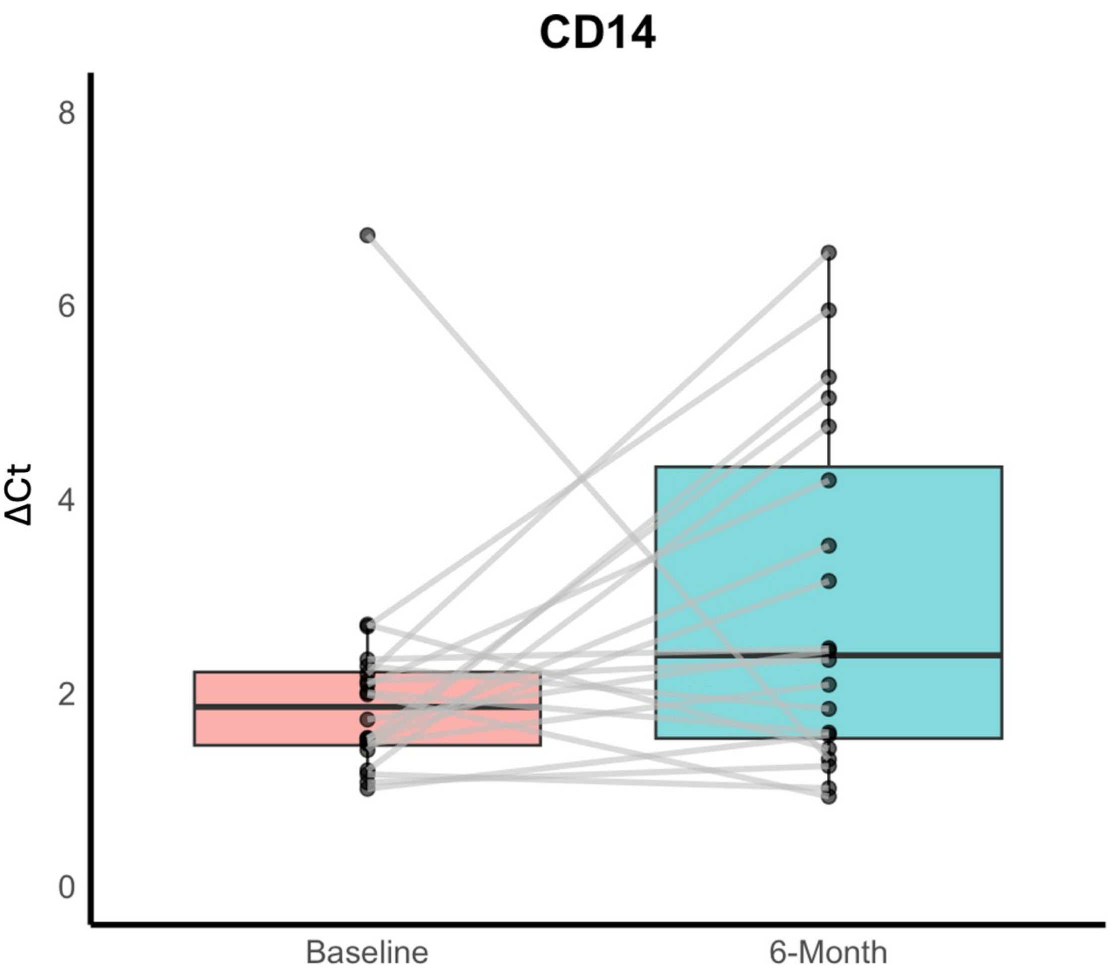

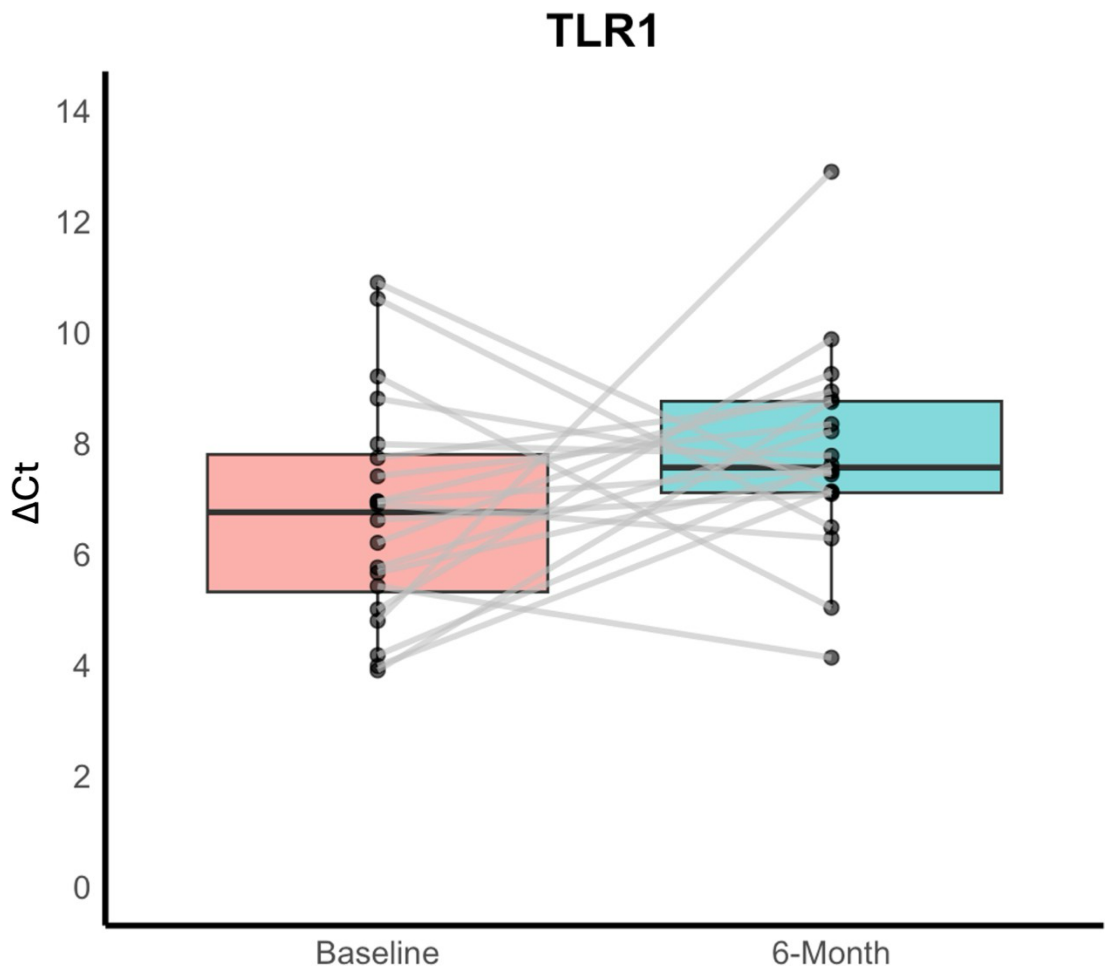
TLR1
ΔCt
14
12
10
8
6
4
2
0
Baseline
6-Month

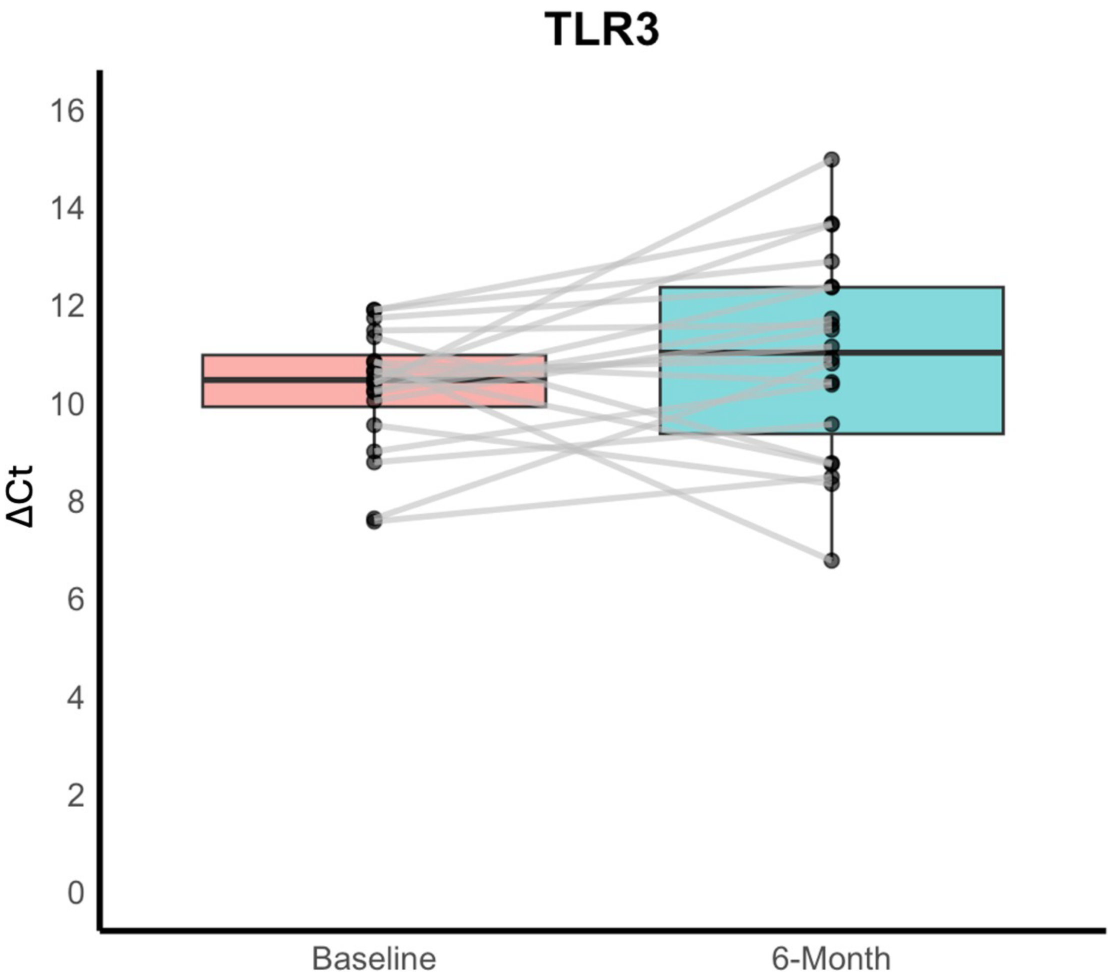

TLR3
16
14
12
10
8
6
4
2
0
ΔCt
Baseline
6-Month

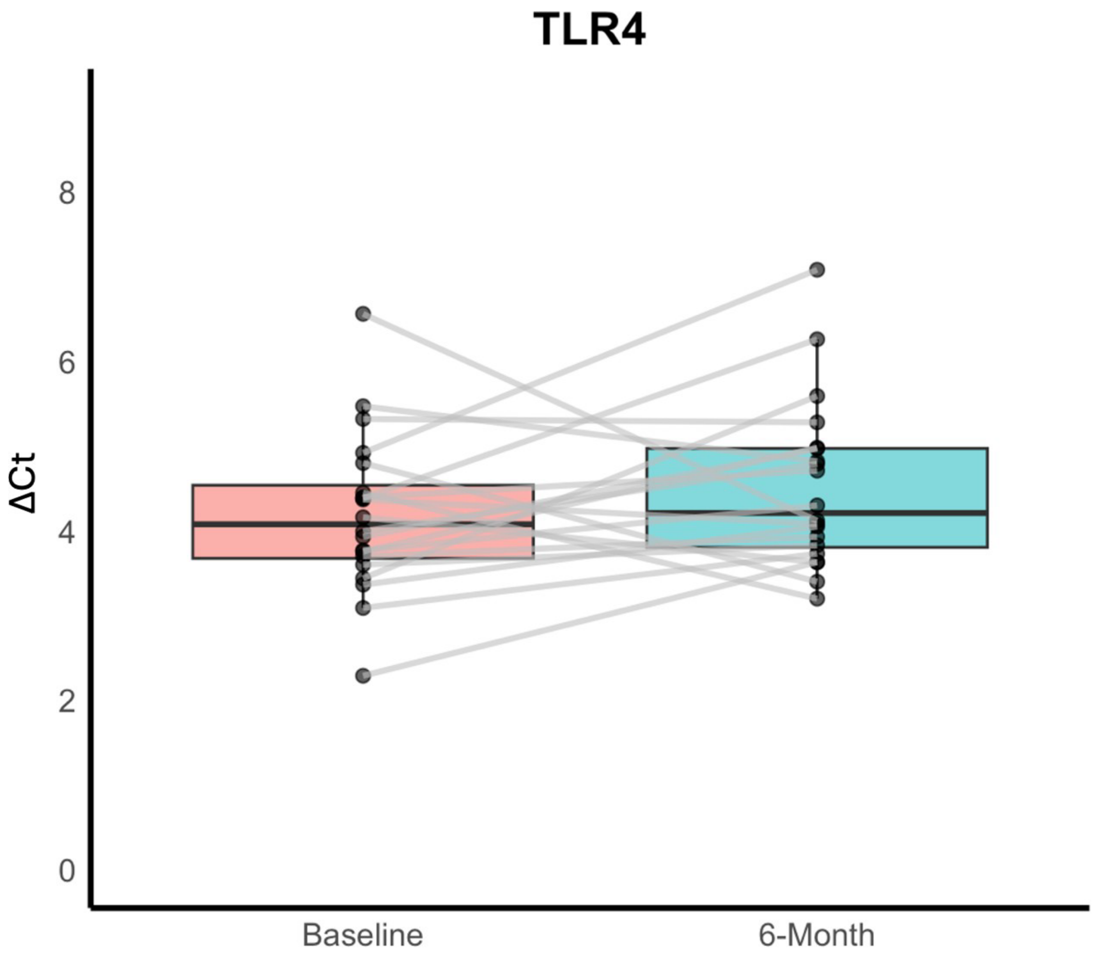

TLR4
ΔCt
8
6
4
2
0
Baseline
6-Month

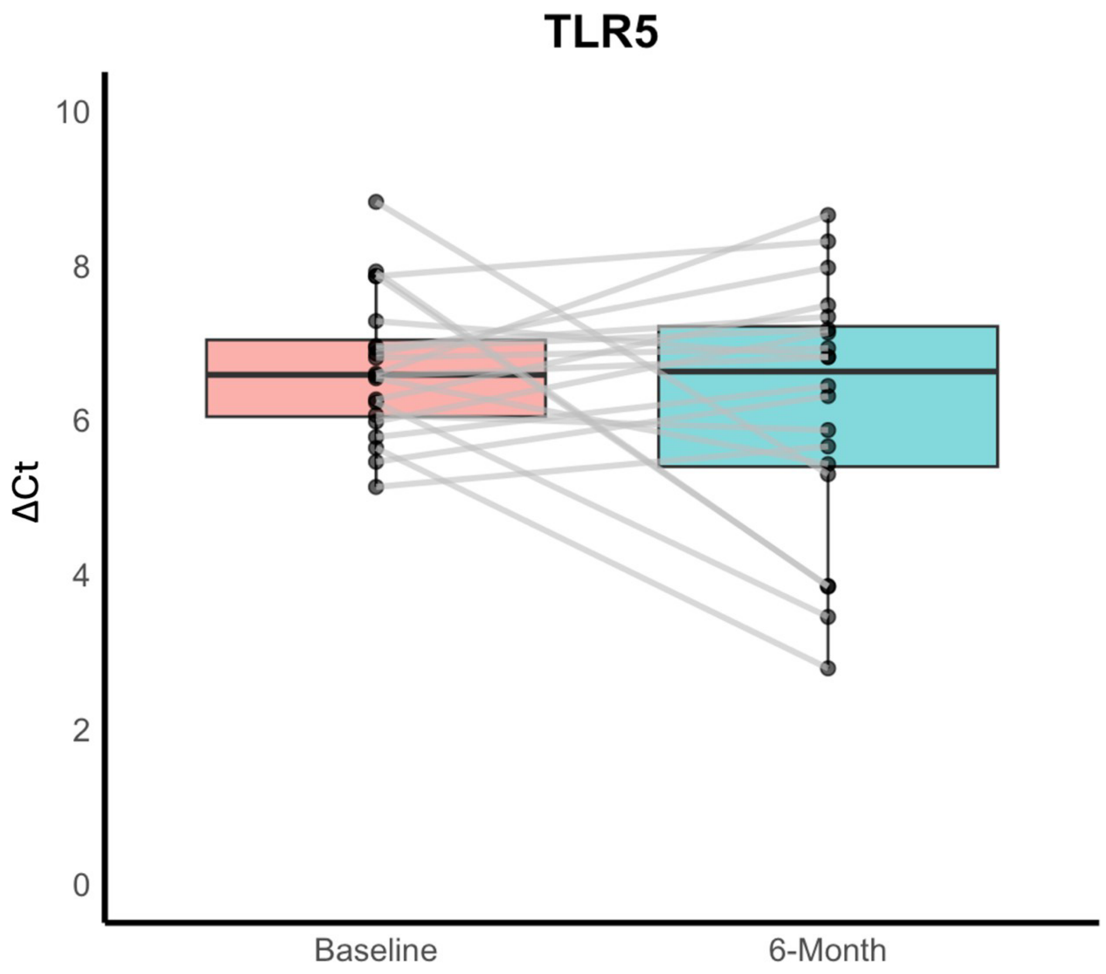
TLR5
10
8
6
4
2
0
ΔCt
Baseline
6-Month

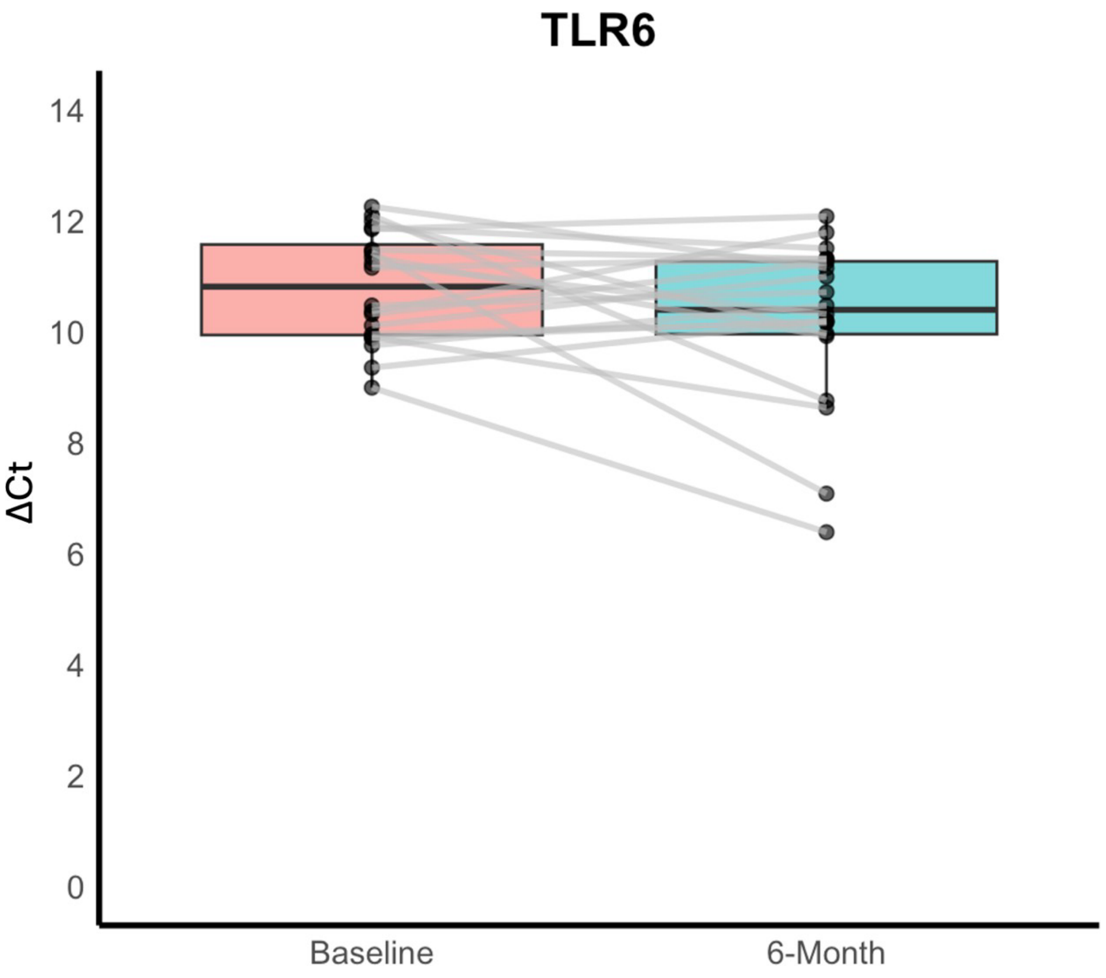
TLR6
14
12
10
8
6
4
2
0
ΔCt
Baseline
6-Month

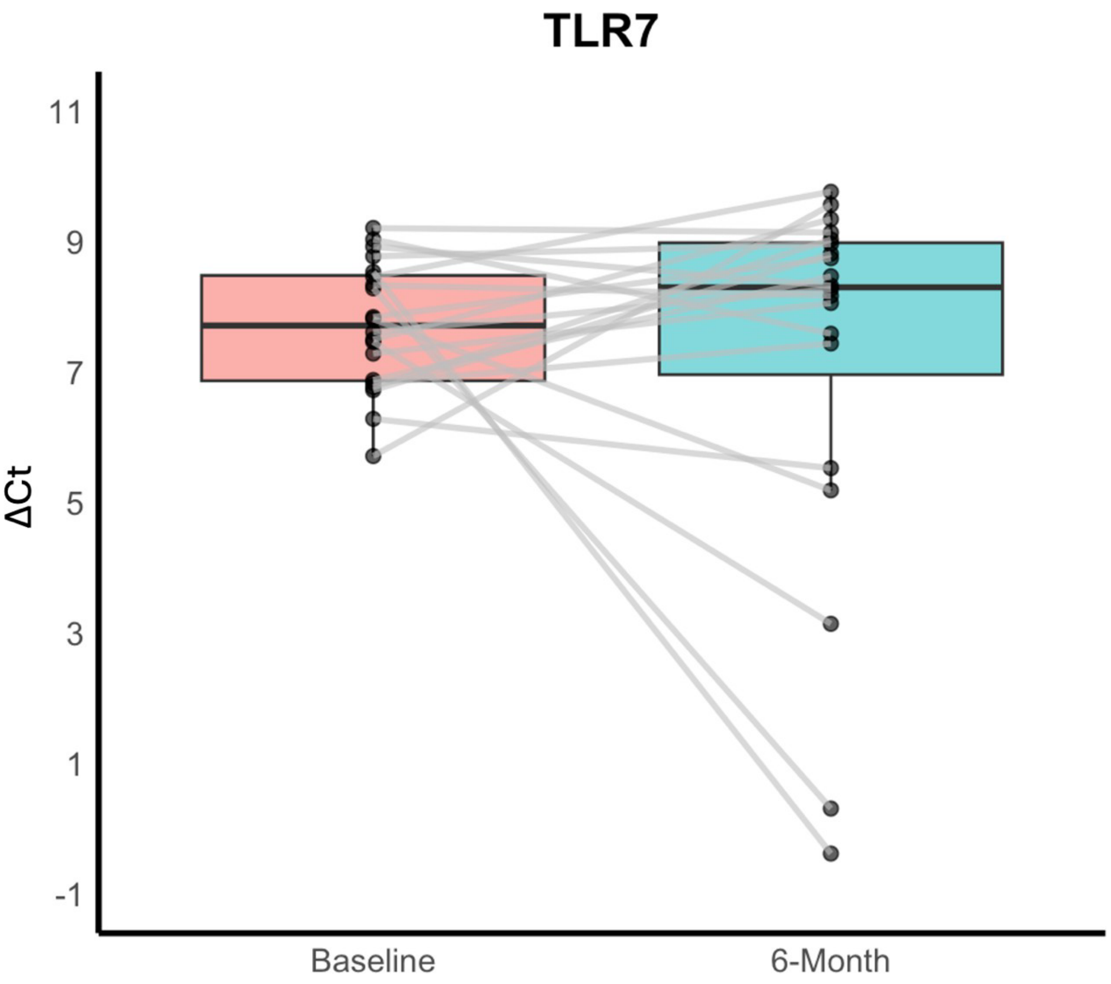
TLR7
11
9
7
5
3
1
-1
ΔCt
Baseline
6-Month
